# The Study of New NiTi Actuators to Reinforce the Wing Movement of Aircraft Systems

**DOI:** 10.3390/ma15144787

**Published:** 2022-07-08

**Authors:** Rafael Braga, Patrícia Freitas Rodrigues, Hélder Cordeiro, Pedro Carreira, Maria Teresa Vieira

**Affiliations:** 1 University of Coimbra, Department of Mechanical Engineering, CEMMPRE, R. Luís Reis Santos, 3030-790 Coimbra, Portugal; rafael.braga@dem.uc.pt (R.B.); teresa.vieira@dem.uc.pt (M.T.V.); 2 Moldes RP, R. José Alves Júnior, 411, 2430-076 Marinha Grande, Portugal; helder.cordeiro@moldesrp.pt; 3 CDRSP-Centre for Rapid and Sustainable Product Development, Polytechnic Institute of Leiria, General Norton de Matos Street, Apart. 4133, 2411-901 Leiria, Portugal; pedro.s.carreira@ipleiria.pt

**Keywords:** actuator, aircraft systems wing, shape memory alloy, NiTi spring, additive manufacturing

## Abstract

Actuators using Shape Memory Alloy (SMA) springs could operate in different mechanical systems requiring geometric flexibility and high performance. The aim of the present study is to highlight the potential of these actuators, using their dimensional variations resulting from the phase transformations of NiTi springs (SMA) to make the movements of the system’s mobile components reversible. This reversibility is due to thermal-induced martensitic transformation of NiTi springs. The transformation promotes the extended and retracted of the springs as the phase changing (martensite–austenite) creates movement in part of the system. Therefore, the phase transition temperatures of NiTi, evaluated by differential scanning calorimetry (DSC), are required to control the dimensional variation of the spring. The influence of the number of springs in the system, as well as how impacts on the reaction time were evaluated. The different numbers of springs (two, four, and six) and the interspaces between them made it possible to control the time and the final angle attained in the mobile part of the system. Mechanical resistance, maximum angle, and the system’s reaction time using different NiTi springs highlight the role of the actuators. Fused Deposition Modelling (FDM)/Material Extrusion (MEX) or Selective Laser Sintering (SLS) was selected for shaping the composite matrix system. A new prototype was designed and developed to conduct tests that established the relationship between the recoverable deformation of the matrix suitable for the application as well as the number and distribution of the actuators.

## 1. Introduction

Aircraft system wing design must satisfy a specific range of flight conditions. Insertion of actuators made from smart materials such as shape memory alloys may overcome the present limitations. Researchers are currently developing new solutions to modify wing design using smart actuators [1]. Mabe J. et al. developed an aircraft system that includes devices that function depending on the performance of an alloy with shape memory [2,3]. The challenge in this approach is to design a structure strong and flexible enough to alter the angle of inclination suitable for the application envisaged. Phase transformations of shape memory alloys (SMAs) can be used to detect variations around them and respond to external stimuli instantly. SMAs have significant recoverable deformation capability and actuating function when SMA structures are submitted to cyclic loading [4,5]. Among the SMAs, NiTi is the most studied due to its specific properties—superelasticity and shape memory effect [6,7]. The change of properties in shape memory alloys (SMAs) can be detected by martensitic transformation. Stress-induced martensite (SIM) promotes superelasticity, and thermal-induced martensitic transformation (TIM) develops the shape memory effect. Therefore, martensitic transformation in NiTi alloys presents both thermal and mechanical hysteresis. Ni content can control the properties. Ni-rich and equiatomic NiTi alloys display the superelastic effect at room temperature and above and Ti-rich NiTi alloys display the shape memory effect above room temperature [8,9]. When the material is cooled from the austenite (A) domain, the martensite (M) starts at M_s_ temperature. The transformation from austenite to martensite is referred to as direct transformation and finishes at martensite M_f_ temperature. When the material is cooled from the austenite (A) domain, the martensite starts its formation at M_s_ and the martensite finishes at M_f_ temperature. On the other hand, when phase M is heated, the austenite phase starts to form at A_s_ temperature. This transformation finishes when the A_f_ temperature is reached. When the material is deformed up to 10% in the martensitic domain, it can retain the deformation, as long as the martensitic stability temperature range is preserved. However, the material starts recovering the original shape when it is heated above A_s_ temperature. When A_f_ is achieved, the shape is recovered by the shape memory effect [5,10].

**Figure 1:**
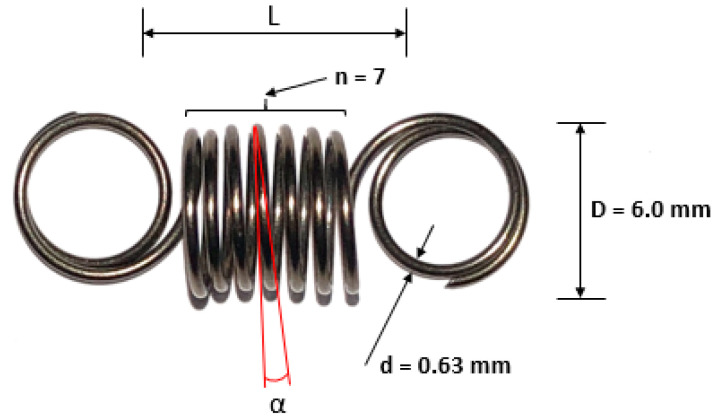
The geometrical design of a NiTi coil spring, thickness (d), outer diameter (D), length (L), number of active coils (n), and initial pitch angle (α).

Different applications of NiTi have contributed to developing new concepts of actuators. The widespread use of NiTi is due to its fatigue behavior and mechanical strength. Depending on the customized application, NiTi is furnished commercially in several forms: spring, wire, thin films, powder particles, and nanoparticles [11]. Many research activities stem from actuator processing, involving NiTi-based materials, especially wire and spring units to construct linear and rotary actuators [12,13]. The typical straight-drawn wire of commercial NiTi has a maximum recovery strain limit of ~4%. A maximum linear strain of ~20% can be achieved using coiled NiTi wires by successively tuning their shape memory properties in the linear coil prototype [14,15], which is the driving force for the movement exerted by the spring on the prototype parts, according to the specified properties of the spring (Figure 1).

The linear actuators activated by shape memory alloys with integrated stroke control are commonly designed and manufactured. The extended and retracted of the actuator are performed by the Joule effect, which can possibly determine the angle to perform in the prototype. One of the main functions of this kind of system applied in the prototype is the possibility to modulate the extension and retraction just by acting on the corresponding SMA springs by heat-induced from an electrical current. As the voltage applied is changed, the extended or retracted springs can be activated in order to set the desired position and overcome the limits of the standard configuration SMA spring [16,17,18]. The aim of this study is to analyze in detail how spring actuators (NiTi) affect the angle of inclination of the composite prototype as a function of the number and distance between the springs.

## 2. Experimental Procedure

**Figure 2:**
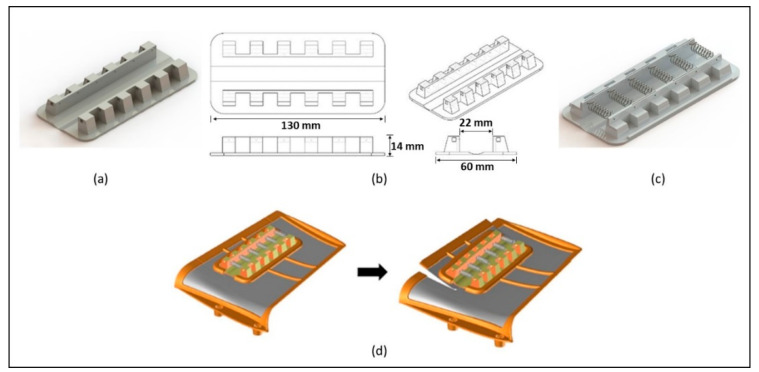
Geometry and assembly of prototype: (**a**) nylon support; (**b**) scheme; (**c**) support with SMA wire and springs; (**d**) aircraft component system before and after SMA actuator effect.

A prototype of the actuator developed to control aircraft system wings constituted by support (nylon) with the incorporation of SMA actuator springs is shown in Figure 2a. The dimensions of the prototype are 130 mm in length and 60 mm in width (Figure 2b). Six SMAs springs (diameter of wire 0.63 mm) were inserted in support specially developed to accomplish the objective to accommodate each one (Figure 2c). Two tiny grooves are cut along the length of the prototype to adjust an SMA wire (on each side) used as an electrical conductor; each SMA spring has contact with the SMA wire (Figure 2c). In the electrical circuit, the current goes through the system of springs to actuate in two, four, six, or eight SMA springs simultaneously. The distance between springs is 10 mm. This system placed in the wings provides control of the structure through different angles. Figure 2d shows the aircraft wing with the actuator.

In this study, the support of prototype production was performed from the CAD project to Material Extrusion (MEX)* and Selective Laser Sintering (SLS). The materials used in each part (support and springs) of the prototype were tested, evaluated, and selected according to their properties.

### 2.1. Material

#### 2.1.1. Spring and Wire

There are several ways to create an electromechanical actuator system. In the present study, the application of the NiTi springs was the solution selected. Therefore, an overview of the properties of springs must be provided. In this case the shape memory alloy NiTi springs (SAES Group-Tensile Spring) have shape memory effect. The dimensions of each spring are, as follows: outer diameter (D) = 6.0 mm, thickness (d) = 0.63 mm, average typical force = 2.0 N and number of turns in the coil (n) = 7 (Figure 1).

**Figure 3:**
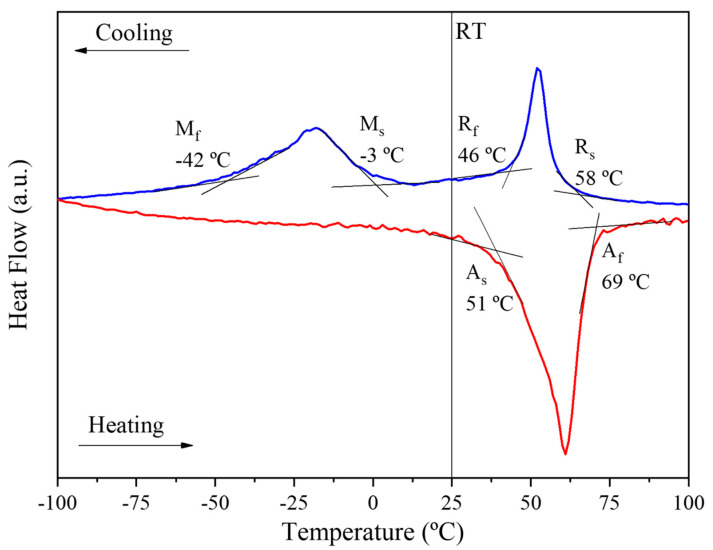
DSC curve of NiTi spring.

The spring has a martensitic phase at room temperature (25 °C), as shown by the Differential Scanning Calorimetry (DSC) curve (Figure 3). Tests were carried out at temperatures ranging from −100 °C to 100 °C under a controlled heating/cooling rate of 10 °C/min. Before analysis by DSC, the specimens were cut and chemically etched (10%HF + 45%HNO_3_ + 45% H_2_O (vol.%)) to remove oxides, as well as the layer deformed by the cutting operation (final mass: ~17 mg). The phase transformation temperatures are: A_s_ = 51 °C and A_f_ = 69 °C; R_s_ = 58 °C; R_f_ = 46 °C, M_s_ = −3 °C; M_f_ = −42 °C. A superelastic NiTi wire (Fort Wayne Metals) with a diameter of 0.5 mm was selected to be used as an electric conductor making contact between the springs, with A_f_ = 27.9 °C [19,20,21,22].

**Figure 4:**
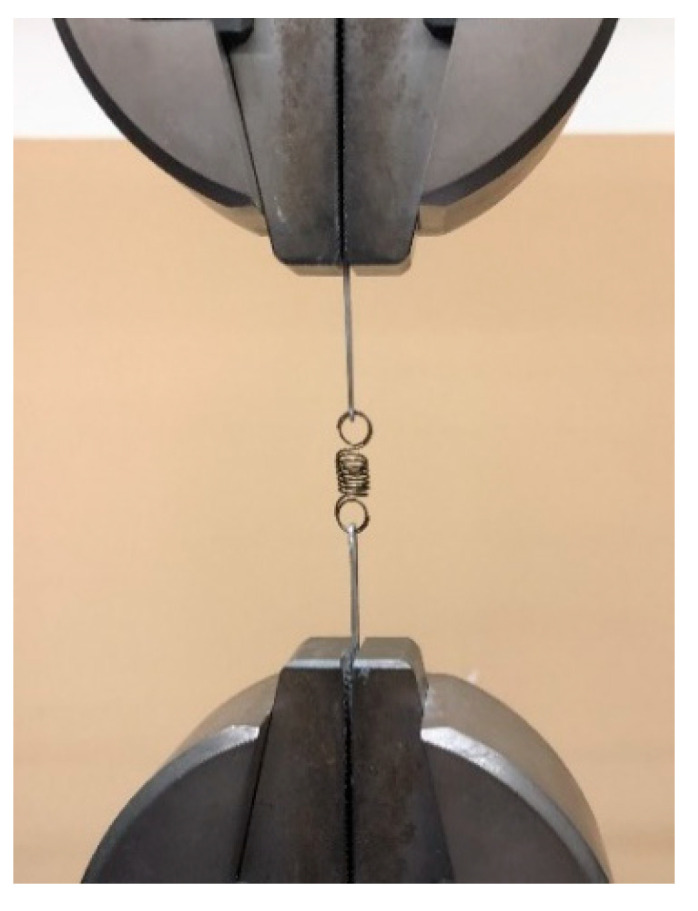
Experimental setup for tensile tests of the NiTi SMA spring.

A Shimadzu Autograph AG-X Universal Tester Machine equipped with a 5 kN load cell was used to test the tensile behavior of the NiTi springs and wires. The SMA spring was connected to the machine using a hook (Figure 4). Then, tensile tests under displacement control at room temperature (25 °C) were performed. After reaching the predefined length (22.0 mm) in the design of the prototype, the displacement was stopped and complete heating/cooling cycles were carried out on the spring. Firstly, the springs were heated (>A_f_ temperature) and then cooled to room temperature (RT) for ten (S10), twenty (S20), and thirty (S30) seconds, in order to simulate the real action of the spring and measure the resistance force imposed by the spring in the test (15 cycles by spring test).

#### 2.1.2. Support

The selection of the material to produce the support resulted from a comparison of Polylactic Acid (PLA), Acrylonitrile Butadiene Styrene (ABS), and Polyamide (PA). The polymeric filament materials PLA (PRIMAVALUE™) and ABS (PRIMASELECT™) are from the supplier PrimaCreator and have 1.75 mm in diameter. Three-dimensional Systems furnished the polymeric powder (Nylon (PA12)) (DuraForm^®^ ProX^®^ PA). Table 1 summarizes the polymeric material properties selected.

The mechanical behavior of the spring support was evaluated by three-point bending and tensile tests (room temperature). The PLA and ABS specimens submitted to three-point bending tests were produced by MEX and the Nylon specimen was manufactured by Selective Laser Sintering (SLS), both with dimensions of 60 × 10 × 2 mm^3^. Three-point bending tests were carried out according to ASTM D790-10, using a Shimadzu Autograph AG-X Universal Tester Machine equipped with a 5 kN load cell, and the TRAPEZIUM X software for data processing, at a displacement rate of 2 mm/min and gauge length of 32 mm.

**Figure 5:**
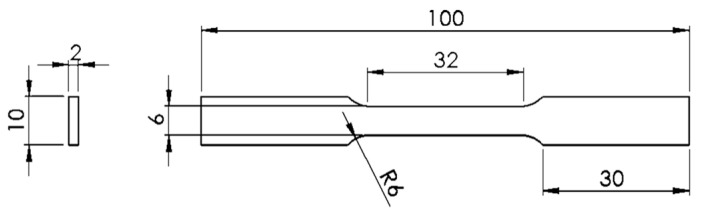
Geometry and dimensions of the specimens for tensile tests.

The specimens submitted to the tensile tests had the geometry shown in Figure 5 with dimensions of 100 × 10 × 2 mm^3^. The tensile tests were performed according to ASTM D 3039, using a Shimadzu Autograph AG-X Universal Tester Machine equipped with a 5 kN load cell, and the TRAPEZIUM X software for data processing, at a load rate of 5 mm/min and a gauge length of 62 mm.

**Figure 6:**
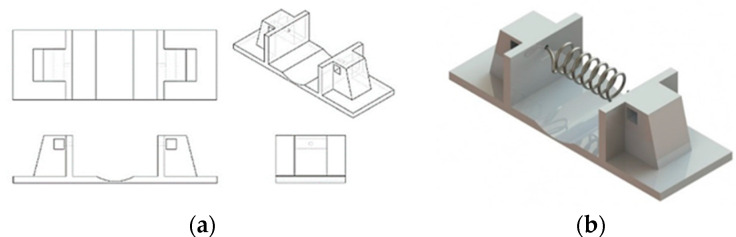
Prototype (one spring): (**a**) schema; (**b**) support with SMA springs.

A prototype was developed using one spring (Figure 6a,b) for the thermal resistance test. In this test, a 9 V DC serial was applied at the springs to evaluate the operating temperature in contact with different materials during ten cycles. The integrity of the polymeric support of the prototype was visually evaluated, and showed no softening or damaging.

### 2.2. Prototypes Fabrication

Two additive manufacturing techniques were selected, to produce the support for the prototype (Figure 2a, MEX and SLS). The dimensions of the support were constant and independent of the number of springs. The MEX parameters were infill density of 40%, gyroid fill patterns for layers, 2 perimeters layers, fill scan pattern angle of 45°, printing speed rate of 50 mm/s, 0.2 mm of layer height, and a printing surface temperature of 215 °C. The equipment used was a Prusa MK3S 3D Printing. The second additive manufacturing technology was supported by a 3D Systems ProX^®^ SLS 6100 3D Printer, where the parameters were as follows: chamber temperature 169 °C, warm-up rate from room temperature 1 °C min^−1^, laser power contour 18W, laser power infilling 60W, laser scan linear speed 237.5 mms^−1^, scan spacing 0.25 mm, layer height 0.1 mm.

The functional characteristics of NiTi springs were studied as actuators in the prototypes in this particular system (Figure 2d). The phase transition temperature of the NiTi spring was attained by a serial connection circuit and a 9 V DC power supply, which can induce the spring to achieve a temperature of between 50 °C and 90 °C. The extended and retracted spring times, with and without induced cooling, and the final angle of inclination reached by the prototype, were measured. The number of springs was changed to evaluate the angle of inclination and the number of springs needed; these variations was tested using two, four, and six springs. In order to guarantee a uniform current, a superelastic NiTi wire makes the electric contact between the springs.

## 3. Results and Discussion

### 3.1. Prototype Details

#### 3.1.1. Actuator

**Figure 7:**
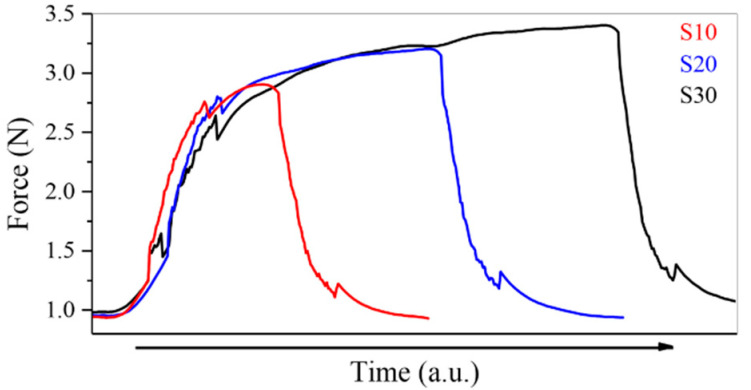
Force vs. time of the thermal cycle of the NiTi spring (tensile test) (10 s (S10), 20 s (S20), and 30 s (S30)).

The results of the NiTi spring tensile tests (Figure 7) highlight the maximum force achieved in the S10, S20 and S30 cycles (heating and cooling cycle times), which corresponds to 10 s, 20 s and 30 s, respectively. This represents the transformation from martensite to austenite through heating. When the spring was subjected to cycles of 10 s (S10), the average force reached was 2.1 N. In longer cycles, the average force reached increased proportionally with time, and was 2.2 N for cycles of 20 s (S20), and 2.3 N in cycles of 30 s (S30) (Table 2).

#### 3.1.2. Support

**Figure 8:**
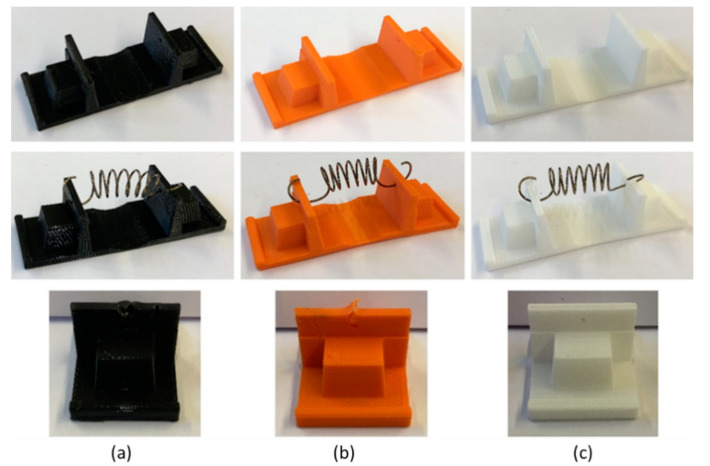
Visual aspect after thermal resistance tests on the supports (one spring): (**a**) PLA; (**b**) ABS; (**c**) PA12.

The visual analyses of the effects of the thermal resistance test using one NiTi spring reveal a significant problem with the PLA after one cycle (Figure 8a) and with the ABS after a few cycles (Figure 8b). When in direct contact with the NiTi spring during heating, imperfections, damage, and some deformation occur at the contact points. These make it impossible to use these materials for supports that work at thermal cycles above the phase transformation temperature of NiTi springs (A_f_ = 69 °C). The PLA shows a service temperature from 45 to 55 °C and ABS between 62–77 °C, which supports the observation of damage. For the degradation temperature when comparing PLA and ABS, the latter one support higher temperatures than the PLA. Using dynamic mechanical analysis (DMA), K. Arunprasath et.al. (2021) observed that a decrease in the degree of crystallinity of both materials (PLA and ABS) increases their strength.

The maximum effective displacement of the actuator support, and consequently of the system, leaded to modify its design creating a zone where it could easily bend without breaking. 

### 3.2. Prototype Tests

**Figure 9:**
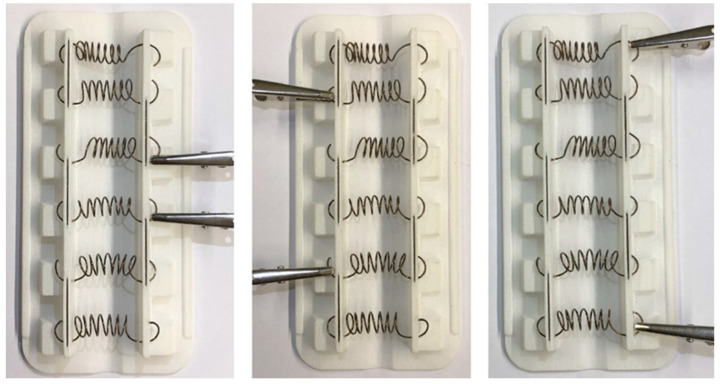
Prototype with six springs and heating system (across different points).

After the selection of the prototype materials, the supports for the prototypes in PA12 were manufactured by Selective Laser Sintering (SLS), and the NiTi springs were assembled to the supports (Figure 9).

**Figure 10:**
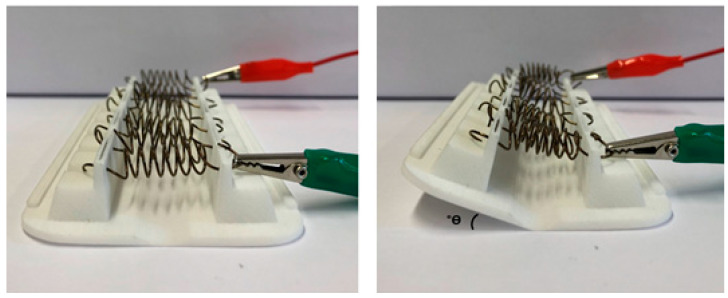
Evaluation of angle of inclination of the prototype.

**Figure 11:**
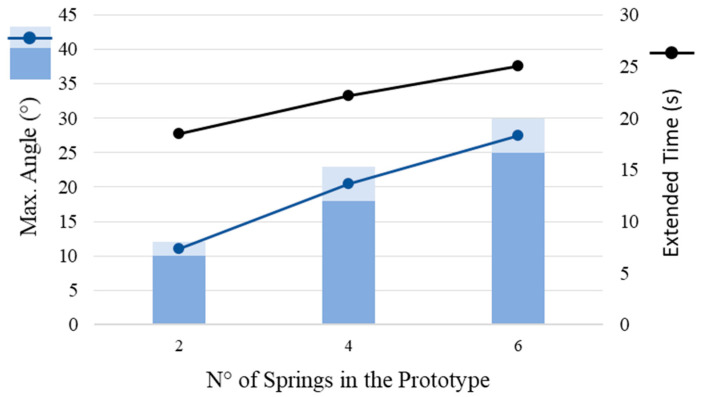
Maximum angle of inclination of the prototype (blue) and extended time as a function of the number of NiTi springs (black) in PA12 support.

The characterization test values for the prototype are summarized in Table 3. The highest angle of inclination of the prototype (Figure 10) was attained for the highest number of springs tested (6). It is possible to vary the angle reached and the extended spring according to the number of springs in the prototype. The retracted time with cooling induction was considerably shorter than without cooling. Figure 11 highlights the linear relationships between the angle of inclination value of the prototype and opening time versus the number of SMA springs.

Nevertheless, it would be interesting to evaluate the prototype’s behavior for different distances between the springs and a more significant number of springs.

## 4. Conclusions

The linear actuation stroke of NiTi (SMA) wire actuators is improved significantly by converting them into coil spring structures (spring). The present study contributes to establishing the main characteristics of a prototype constituted by spring actuators based on NiTi (SMA) with unique thermo-mechanical performances for wing aircraft system. A PA (nylon) support produced by additive manufacturing was selected because it is strong enough for the forces applied and resistant to the temperatures developed during the application.

The main characteristics for the industrial application of NiTi actuators are as follows:

− The maximum inclination angle of the support is a linear function of the number of springs;
− Cooling of the prototype has significant importance regarding prototype recovery time;
− The maximum inclination angle of the prototype is a linear function of the temperature imposed on the spring.

## Figures and Tables

**Table 1:** Temperature, fracture toughness, and Young’s modulus of PLA, ABS, and PA12 (CES 2013 Edupack).

Material	Service Temperature (°C)	Fracture Toughness (MPa.m^1/2^)	Young’s Modulus (GPa)	Tensile Strength (MPa)
PLA	45–55	0.70–1.10	3.45–3.83	48.0–60.0
ABS	62–77	1.90–2.10	2.00–2.90	30.0–50.0
PA12	90–130	3.32–3.66	1.33–1.65	58.5–71.5

**Table 2:** Maximum force function of the thermal cycle (NiTi springs).

Cycle Heating/Cooling	Maximum Force (N)
S10	2.1
S20	2.2
S30	2.3

**Table 3:** Extended and retracted time with and without induced cooling and maximum angle of inclination.

Number of Springs	Extended Time (s)	Retracted Time with Induced Cooling-Air (s)	Retracted Time without Induced Cooling-Air (s)	Maximum Angle (°)
2	~19	20–35	75–90	10–12
4	~23	20–35	75–90	18–23
6	~25	20–35	75–90	25–30
